# A profile approach to physical activity levels: what’s intensity got to do with reasons and motives for exercise?

**DOI:** 10.1186/s12889-024-20449-1

**Published:** 2024-10-29

**Authors:** Vanessa M. Martinez Kercher, Damon Burton, Kyle A. Kercher, Kathleen N. Heeter, Julia Brunnemer, Janette M. Watkins, Andrew C. Pickett, Michael A. Pickering

**Affiliations:** 1 Department of Health & Wellness Design, Indiana University, Bloomington, IN USA; 2 Department of Movement Sciences, University of Idaho, Moscow, ID USA; 3 Department of Kinesiology, Indiana University, Bloomington, IN USA; 4 Department of Applied Health Science, Indiana University, Bloomington, IN USA

**Keywords:** Goals, Reasons to exercise, Behavioral regulation, Self-determination, Cluster analysis

## Abstract

**Background:**

Despite the well-known benefits of physical activity (PA), non-communicable disease and premature mortality rates among adults continue to rise. The relationship between adults’ goals and exercise-specific motivation on the type of PA intensity one engages remains unclear. The purpose of this study was to identify physical activity (PA) profiles based on frequency and intensity (i.e., levels of PA) in an adult sample. A secondary purpose was to examine how the PA profiles differ on the reasons people have for exercising and behavioral regulation.

**Methods:**

A Cross-sectional survey was conducted with 1,169 (46.8 ± 16.7 years) participants solicited from a hospital-affiliated wellness center, social media promotions, and a research volunteer registry. The International PA Questionnaire (IPAQ) was used to determine frequency, intensity, and time spent engaging in PA. Additionally, the Reasons to Exercise (REX-2) scale, the Behavioral Regulation in Exercise Questionnaire-3 (BREQ-3), and demographics were assessed. K-cluster analyses were performed to identify profiles based on PA levels using the IPAQ guidelines. Multivariate analysis of variance (MANOVA) was used to assess profile differences.

**Results:**

Five distinct PA clusters were derived, and defined as: a Low, Walking, Moderate Intensity, High Intensity, and Sitting cluster (p < .001). These clusters differed significantly (*p* < .001) from each other with respect to motivation, the reasons adults have for exercise, and PA levels.

**Conclusion:**

The results from this study support the important role of psychological factors such as motivation and reasons for exercise on behavioral outcomes (i.e., physical activity). For future research investigating adults PA- related behaviors, whether it be on adults starting a new exercise program or for PA maintenance, it may be beneficial to develop programs that encourage participants to reflect on the reasons they identify as important for exercising, and how such reasons contribute to their overall PA engagement behaviors.

**Supplementary Information:**

The online version contains supplementary material available at 10.1186/s12889-024-20449-1.

## Introduction

The mental, physical, and holistic health benefits associated with engaging in moderate-to-vigorous intensity physical activity (PA) are well-supported [1]. However, less than 1 in 4 adults in the US engage in the recommended 150 min of moderate intensity or 75 min of vigorous PA per week [1]. According to the Sedentary Behavior Research Network, PA refers any bodily movement produced by skeletal muscles that require energy expenditure [2]. The concept of PA encompasses various forms, such as walking, running, and cycling and can be classified by intensity (e.g., light/walking, moderate, vigorous) mode, and domain (e.g., occupational, recreational). However, sedentary behavior—characterized by low energy expenditure (≤ 1.5 METs) in a sitting or reclining poster—remains on the rise, further complicating efforts to promote healthier habits [2]. This trend places many adults at increased risk for non-communicable diseases and premature mortality [3]. While PA participation represents a challenging health behavior within a complex, multilevel system, there is a considerable variety and heterogeneity within the broad category of physical activities. In this study, the term “physical activities” (PAs) used throughout the paper refers specifically to the various types and intensities of PAs as defined above within the SBRN definitions by Tremblay and colleagues [2].

Understanding patters of PA engagement remains a significant area of research, particularly regarding how different PA behaviors are associated with individual motivations. In motivation research, both “what” people pursue (i.e., goal contents) and “why” they pursue it (i.e., behavior regulation) are important for understanding PA behaviors [4, 5]. According to Self-Determination Theory (SDT), motivation is not a singular construct but exists a long a continuum, ranging from intrinsic (i.e., integrated, identified forms of autonomous motivation) to extrinsic (controlled forms of motivation-external and introjected) forms of regulation [6–8]. Autonomous motivation, which stems from enjoyment, whereas controlled motivation, driven by external pressures, can negatively impact PA adherence [6–8]. Adults who exercise for appearance-related reasons, for example, may experience their goals as externally controlled, leading to diminished PA participation. In contrast, goals related to personal challenge or affiliation are more often autonomously motivated and promote sustained PA involvement [5]. Strong support [6, 9] highlights how different goals for engaging in PA often naturally co-exist in the same person, some being more intrinsic, and others less so. Therefore, it is the relative preponderance of certain types of goals versus others that is thought to determine more or less favorable outcomes related to PA engagement. While the combination of multiple goals (intrinsic or extrinsic) findings are not new and there has been considerable research done to understand PA goals and motivation, the classification system of PAs to understand psychological tendencies may provide insight into PA patterns that can be supported or challenged in what we know about motivation through behavioral regulation [5, 8, 9]. Several classification systems have been prosed for the purpose of grouping, differentiating, and organizing PAs [10, 11]. PA can be classified by context, such as leisure, commuting, household, and work [12]. However, reporting of PA categories varies greatly depending on what measure is used to define and/or describe engagement in PAs. For instance, type of PA is one approach that may commonly be classified into sports and exercises, and each of them further subdivided [12, 13]. The intensity of energy expenditure can also classify PA as light, moderate, or vigorous [12, 14]. PAs can further be divided into aerobic and anerobic, depending on the metabolic requirements [12]. Categories can also be used to break PA into low, moderate, or highly active categories. The classification systems for PAs based on psychological properties, such as motivation, is an area that continues to be explored but not clearly understood. The categorization of PAs through the lens of goals can be valuable because it may naturally organize and anchor the different PAs categories into universal psychological tendencies.

While previous research has examined the relationships between PA and motivation, much of the work has been limited by the way PA is categories—often focusing on specific types, modes, or domains of activity [6, 14]. Less attention has been given to how profiles based on the combination of PA frequency and intensity are associated with motivation and goals. Understanding how different PA profiles—defined by specific combinations of activity frequency and intensity—relate to motivational factors may provide new insights into PA engagement. For instance, do adults who engage in vigorous-intensity activities, such as running or high-intensity interval training, have different profiles compared to those who engage in moderate-intensity activities like walking? Furthermore, can these profiles predict not only the reasons people exercise but also the underlying behavioral regulation that sustains PA?

The goals, which we will also refer to as reasons, adults focus on in their PA efforts are a guide to understanding patterns of engagement [15]. These goals can initially drive PA behavior, and positive experiences during PA can, in turn, reinforce or shift these goals and motivations.

Identifying patterns of PA have been investigated in individual and team sports [16], leisure PA [17], mode [8], frequency [18], and intensity [10]. Early research [17] in leisure PA found that highly active and moderately-high active adults differed from their less active counterparts by placing more importance on health-related goals (i.e., health fitness and mental health), whereas highly active adults differed from moderately active and less active adults by placing more importance on achievement-related goals (i.e., feel good, task, and outcome incentives). Additionally, adults’ participating in fitness-related activities focused on appearance and social related goals for engaging in PA compared to those who engage in more walking [19]. In more recent work [10], findings indicated that participants engaging in vigorous PA were represented with higher mean scores in interest/enjoyment, competence, and social goals, whereas those engaging in moderate PAs scored higher on appearance and fitness and health-focused goals. To expand on previous work [6, 20, 21], understanding the impact of additional goals may provide researchers and practitioners with more insight into understand differences in PAs.

Previous research has explored PA categories for identifying differences in *goals* for engaging in different PA (e.g., individual sports, collective sports, exercises, and body/movement practices) using a variety of different instruments (MPAM—Motives for Physical Activity Measure, MPAM-R—Motives for Physical Activity Measure-Revised, and PALMS—Physical Activity Leisure Motivation Scale) ranging from three or more goals [16, 22, 23]. Many of these studies’ findings have contributed greatly to our knowledge, and to continue to contribute to such findings, the present study addressed three gaps in the literature. First, while some previous studies have utilized motivation profiles to explore the impact of motivation and PA related outcomes [18, 23, 24] few have explored classification systems of PA solely grouped based on patterns of PA frequency and intensity (e.g., walking, moderate, vigorous PA intensity). Second, due to the number of different approaches to PAs (e.g., type, mode, intensity, and/or not defined), reported findings on PA outcomes vary considerably, making it more difficult to create a classification system of PA to understand motivational tendencies. Third, due to the limited scope of instruments employed, numerous studies do not involve the whole spectrum of potential goal pursuits to explore patterns based on PAs.

The terminology, reasons for exercise, and goals are used interchangeably throughout this study. To our knowledge, few studies have attempted to identify and classify PA into profiles based solely on PA (frequency and intensity), and fewer have explored whether such PA profiles vary in the type of goals and sources of behavioral regulation (e.g., external, introjected, identified, integrated, and intrinsic motivation) hypothesized to influence PA frequency patterns. Therefore, the objectives of this study was threefold: (a) determine whether adults can be differentiated by examining their PA profiles (e.g., unique combinations of PA type/intensity engagement classification), (b) to understand the relationship between PA profile patterns on type of goals adult’s identify as important for engaging in PA, and (c) examine the relationship between these PA clusters groupings on the underlying sources of motivation (i.e., behavioral regulation) and goals. We hypothesize that distinct PA profiles exist and that these profiles are associated with different motivational patterns and goals. Specifically, we aim to address the following research questions: 1) What PA profiles based on PA levels (i.e., PA type/intensity) exist among adults? (b) Do these PA profiles exhibit PA levels that result in unique patterns of goals (i.e., reasons for exercise)? And (c) What is the relationship between PA levels, reasons for exercise, and the quality of motivation (e.g., relative autonomy index score)? By addressing these questions, this study aims to provide a clearer understanding of how variations in PA engagement influence motivational factors and exercise behaviors, ultimately contributing to the development of more effective interventions for promoting sustained PA.

## Methods

### Participants

A total of 1,169 responses (ranging from 18 to 87 years of age) included participants from a hospital-affiliated wellness center (*N* = 73), social media promotion interactions (*N* = 236), and a research volunteer registry (*N* = 860). Participants consisted of 478 males (40.9%), 690 females (59.0%), and one respondent (0.1%) who did not indicate gender. The average adult was middle-aged (*M* = 46.8 years; *SD* = 16.7).

## Procedures and design

Upon receiving ethical approval from the Institutional Review Board at the first author’s university, this study employed a cross-sectional, survey-based design. The survey was administered to a convenience sample of adults aged 18 and older, recruited from three main sources: a hospital-affiliated wellness center, social media promotions, and ResearchMatch (a research volunteer registry). An online survey with a combination of multiple-choice and Likert scale questions was developed through Qualtrics and administered to the three convenience samples of adults aged 18 years of age and older recruited to participate in the survey. For in-person recruitment, a table was set up in the hospital-affiliated wellness center where participants were asked to complete an 8–10-min survey electronically on a tablet. The wellness center was selected for recruitment as it serves a wide range of individuals with varying demographics, increasing the likelihood of a diverse sample. For both social media promotions and the research volunteer registry (i.e., ResearchMatch), participants were recruited via email invitations and through social media promotions. with the URL to access the online survey. Recruitment efforts were targeted to adults 18 + across diverse geographic regions and backgrounds. Individuals who expressed interested were directed to complete the survey through Qualtrics. The inclusion criteria of this study required participants to be aged 18 years or older and able to read and understand English. Participants were excluded if they did not provide informed consent or failed to complete the survey. Participation in this study was voluntary. Informed consent was acquired from all participant volunteers. To maintain study confidentiality, all responses were de-identified.

### Instrumentation

#### Reasons for Exercise Scale

The Reasons to Exercise Scale (RE_X_-2) scale contains 9 factors represented by 36 items: (a) fitness, (b) competition; (c) solitude; (d) social; (e) appearance; (f) weight management; (g) health concerns; (h) mood enhancement; and (i) preventative health. Scale items contained a standardized stem (i.e., “To you, how important is this reason for exercising and/or being physically active?”) in conjunction with content statements analyzed using a 6-point Likert scale, ranging from 1 (not at all important) to 6 (extremely important). Throughout the development of the Reasons to Exercise Scale, participants were asked to provide their personal "reasons" for exercise rather than identifying specific goals [25]. This more inclusive language was used so participants were able to better resonate to and respond about their deeper "why" behind being physically active. Therefore, in this study, "reasons" for being physically active was used interchangeably to promote adult’s identifying more informal goals [25].The reliability and validity of the RE_X_-2 has demonstrated acceptable psychometric properties with adults [25]. Cronbach’s alphas in this study ranged from 0.84 to 0.94 (see Supplemental Table 1).

**Table 1:** Descriptives and Correlations for Physical Activity Intensity Levels, Reasons to Exercise Subscales, and RAI Scores

	**Variables**	**1**	**2**	**3**	**4**	**5**	**6**	**7**	**8**	**9**	**10**	**11**	**12**	**13**	**14**
**PA Levels**	1. Vigorous PA	–													
	2. Moderate PA	.30	–												
	3. Walking PA	.11	.22	–											
	4. Sitting	-.15	-.18	-.09	–										
**Reasons for Exercise**	5. Mood enhancement	.31	.19	.09	-.12	–									
	6. Solitude	.27	.14	.09	-.13	.62	–								
	7. Social	.29	.15	.07	-.13	.48	.39	–							
	8. Fitness	.32	.13	.11	-.13	.56	.36	.42	–						
	9. Weight management	**.01**^**#**^	**-.05**^**#**^	**-.01**^**#**^	**-.01**^**#**^	.19	.15	.15	.26	–					
	10.Preventative health	.16	**.06**^**#**^	**.04**^**#**^	-.10	.39	.26	.23	.57	.30	–				
	11. Appearance	.12	**.04**^**#**^	**.03**^**#**^	**-.02**^**#**^	.40	.24	.23	.42	.59	.30	–			
	12. Health concerns	**-.04**^**#**^	**-.01**^**#**^	**.04**^**#**^	-.06	.06	.11	.10	.17	.30	.24	**.05**^**#**^	–		
	13Competition	.30	.17	.08	-.11	.33	.33	.51	.38	**.04**^**#**^	.10	.27	-.06	–	
	14. RAI Score	.42	.24	.10	-.18	.64	.44	.42	.49	-.08	.30	.15	-.12	.31	–
	*Mean*	157.4	142.8	246.2	302.1	4.2	3.2	2.7	4.3	3.9	4.7	3.9	3.2	2.0	15.1
	*SD*	163.2	140.1	253.8	161.2	1.3	1.4	1.2	1.0	1.3	1.0	1.3	1.4	1.2	6.2

All correlations significant at *p* ≤ 0.05 unless otherwise bolded and ^#^ *p* > 0.05. SD = standard deviation; RAI = Relative Autonomy Index score (i.e., motivation quality); PA = Physical Activity. PA levels reprsent total accumulated minutes per week

#### Physical activity levels

The International Physical Activity Questionnaire (IPAQ) short form is used to obtain comparable estimates of total PA minutes/week. Total scores require duration (in minutes) and frequency (days) from activities such as sitting, walking, moderate-intensity and vigorous-intensity activity to provide a measure of an individual’s PA during the most recent seven-day period [26]. The total accumulated minutes per week was estimated for each variable including vigorous-intensity PA, moderate-intensity PA, walking, and sitting. Reports on the IPAQ’s ability to measure PA levels have demonstrated a test–retest reliability coefficient of 0.80 among 18 to 65 years in diverse settings [26].

#### Behavioral regulation

The Behavioral Regulation Exercise Quesstionaire-3 (BREQ-3), derived from Deci and Ryan’s SDT continuum of controlling to autonomous forms of motivation, measures external, introjected, identified, integrated, and intrinsic forms of exercise regulation using a 5-point Likert scale, ranging from 0 (not true for me) to 4 (very true for me) [5]. Items of the BREQ-3 include, but are not limited to highlighting external (e.g., I exercise because other people say I should), introjected (e.g., I feel guilty when I don’t exercise), identified (e.g., I value the benefits of exercise); integrated (e.g., I consider exercise part of my identity), and intrinsic regulation (e.g., I exercise because it’s fun) [5]. The reliability and validity of the BREQ-3 has demonstrated acceptable psychometric properties with adults [27]29. A single score is calculated by summing each subscale score, creating a list outlining the degree to which respondents feel autonomous (i.e., higher motivation quality). ‘Relative autonomy index’, or RAI, is calculated from the summation. Cronbach’s alphas for each subscale in this study ranged from 0.81 to 0.94 (see Supplemental Table 1).

#### Physical activity demographics and background

Participants were asked to self-report their age, gender, ethnicity, highest level of education, and experience engaging in PA/exercise. Age was reported as a continuous variable, assessed using a sliding scale. Participants were asked to report their gender as “male”, “female”, or “prefer not to answer”. PA experience questions included the number of years being physically active (open-ended), whether respondents participated in sports over their lifetime (categorical), and the highest level of sports participation (i.e. youth sport to Olympic sport).

### Data analysis

The Statistical Package for the Social Science (SPSS, Version 29) was used to analyze the data. Screening for missing values, outliers, and normality were performed before analysis. Descriptive statistics for all outcome variables and participant demographics (i.e., age, gender, ethnicity, sports participation, and education background) were measured. Alpha coefficients were assessed for all psychometric data. Pearson correlations were used to investigate the relationship between all outcome variables. Correlations were categorized as weak, moderate, and strong (*r* = 0.10, 0.30, and 0.50, respectively) [28]. No data were imputed since less than 5% of the data were missing at random [29].

Distinct PA profiles were developed using cluster analyses. We performed two-step hierarchical clustering [30, 31] to examine initial cluster solution profiles using the classified PA groupings (e.g., sitting behavior, walking, moderate and vigorous PA intensity) using the IPAQ guidelines [26]. Ward’s method was employed [32] to provide guidance of the number of clusters represented in the data and to reduce the within cluster differences. In the second step, PA scores were changed into z-scores prior to conducting the cluster analysis. Then, following Ward’s method, a non-hierarchical k-means cluster procedure was performed to assess the cluster algorithm. From prior research by K Siddiqui [33], because there is not a rule of thumb for cluster analysis, based on the same dimensionality problem we followed the factor analysis rule of thumb, where our minimum sample size should be 250; thus, the sample was powered enough for the study.

Descriptive statistics were used for calculating means and standard deviation for age, gender, ethnicity, highest level of sports participation, and education background for each cluster. To interpret the characteristics of each cluster, multivariate analysis of variance (MANOVA) was conducted to confirm whether significant differences tested between the cluster profiles across the reasons for exercise and behavior regulation outcome variables (i.e., external, introjected, identified, integrated, intrinsic regulation, and overall RAI scores for motivation quality). Alpha levels of 0.05 were used with partial eta squared (partial *η*^2^) values reported using Cohen’s [34] guidelines for effect size interpretation. Analysis of variance (ANOVA) with post-hoc comparisons and Bonferroni corrections were used when Wilks’ lambda was significant.

## Results

### Preliminary analyses

Descriptive and correlation data for all dependent variables can be found in Table 1 and Supplemental Table 1.

Participants engaged in a total (minutes/week) of 157.4 vigorous PA, 142.8 moderate PA intensity, and 246.2 in walking. Vigorous, moderate, and walking PA total minutes/week were positively and significantly associated with RAI scores (ranging from *r* = 0.10 to 0.42, *p* < 0.01) and negatively associated with sitting behavior (*r* = -0.18; see Table 1). Total vigorous PA intensity (total minutes/week) was positively and significantly associated with 7 reasons for exercise subscales: fitness, mood enhancement, competition, social, solitude, preventative health, and appearance (ranging from *r* = 0.12 to 0.32; see Table 1), but not associated with weight management and health concerns subscales. Moderate PA and walking were positively, albeit weakly, associated with five of the reasons for exercise subscales: mood enhancement, solitude, social, and competition (ranging from *r* = 0.07 to 0.19; see Table 1).

### Cluster results for PA intensity levels

The final cluster analysis revealed a 5-profile solution for examining PA that was deemed the optimal model based on empirical and conceptual predictions from PA literature [4, 5]. K-means produced similar 5-cluster solution means. Cluster sizes, descriptive statistics, and mean comparison of the PA profiles are shown in Table 2 and Table 3.

**Table 2:** Participants Demographics Distribution across the five PA profiles

5-PA Profiles	Cluster 1	Cluster 2	Cluster 3	Cluster 4	Cluster 5	Total
	*N* = 403	*N* = 143	*N* = 127	*N* = 116	*N* = 380	1,169
	**Low PA**	**High PA**	**Moderate PA**	**Walking/** **Walkers**	**Sitting/** **Sitters**	
Age in years (M ± SD)	48.9 ± 17.1	47.5 ± 16.3	49.9 ± 17.8	43.1 ± 17.1	44.1 ± 15.4	
Gender (Male/Female) %	M: 35.5% F: 64.5%	M: 58.7% F: 41.3%	M: 45.7% F: 54.3%	M: 44.8% F: 55.2%	M: 37.1% F: 62.6%	
**Ethnicity N (%)**						
African American	9 (27.3%)	4 (12.1%)	6 (18.2%)	2 (6.1%)	12 (36.4%)	33
American Indian/Alaska Native	2 (40.0%)	1 (20.0%)	1 (20.0%)	1 (20.0%)	0	5
Asian	8 (27.6%)	3 (10.3%)	2 (6.9%)	2 (6.9%)	14 (48.3%)	29
Hispanic	42 (38.9%)	17 (15.7%)	8 (7.4%)	17 (15.7%)	24 (22.2%)	108
Multiracial	9 (33.3%)	6 (22.2%)	1 (3.7%)	3 (11.1%)	8 (29.6%)	27
Native Hawaiian/Pacific Islander	0	0	0	1 (50%)	1 (50%)	2
White	333 (34.5%)	112 (11.6%)	109 (11.3%)	90 (9.3%)	321 (33.3%)	965 (100%)
**Gender N (%)**						
Male	143 (29.9%)	84 (17.6%)	58 (12.1%)	52 (10.9%)	141 (29.5%)	478 (41%)
Female	260 (37.7%)	59 (8.6%)	69 (10%)	64 (9.3%)	238 (34.5%)	690 (59%)
Prefer not to say	0	0	0	0	1(100%)	1 (100%)
**Highest Sports Level N (%)**						
No sport	153 (34.9%)	38 (8.7%)	44 (10.0%)	46 (10.5%)	157 (35.8%)	438
Youth sport	13 (46.4%)	4 (14.3%)	3 (10.7%)	3 (10.7%)	5 (17.9%)	28
Middle/Junior sport	21 (41.2%)	4 (7.8%)	4 (7.8%)	5 (9.8%)	17 (33%)	51
High school sport	77 (32.9%)	29 (12.4%)	28 (12.0%)	23 (9.8%)	77 (32.9%)	234
Intramural sport	86 (35.1%)	38 (15.5%)	23 (19.4%)	22 (9.0%)	76 (31.0%)	245
College sport	39 (28.9%)	22(16.3%)	19 (14.1%)	16 (11.9%)	39 (28.9%)	135
Master level sport	11 (37.9%)	6 (20.7%)	5 (17.2%)	1 (3.4%)	6 (20.7%)	29
Professional sport	2 (28.6%)	2 (28.6%)	1 (14.3%)	0	2 (28.6%)	7 (100%)
**Education N (%)**						
Some school, no high school	1 (50%)	0	0	0	1 (50%)	2
High school diploma	146 (36.0%)	59 (14.5%)	38 (9.4%)	31 (7.6%)	132 (32.5%)	406
Associate degree	41 (35.0%)	9 (7.7%)	15 (12.8%)	16 (13.7%)	36 (30.8%)	117
Bachelor’s Degree	45 (47.4%)	6 (6.3%)	9 (9.5%)	13 (13.7%)	22 (23.2%)	95
Master’s Degree	34(31.8%)	16 (15.0%)	10 (9.3%)	8 (7.5%)	39 (36.4%)	107
Doctoral Degree	136 (30.8%)	53(12.0%)	55 (12.4%)	48 (10.9%)	150 (33.9%)	442 (100%)

Data are reported as either mean ± SD or N (%). PA = Physical Activity

**Table 3:** MANOVA results comparing five PA profiles for reasons to exercise, motivation regulation, and PA categories

**5-PA Profiles**	Cluster 1		Cluster 2		Cluster 3		Cluster 4		Cluster 5			
	Low PA		High intensity PA		Moderate Intensity PA		Walking PA		Sitting			
	*N* = 403		*N* = 143		*N* = 127		*N* = 116		*N* = 380			
**Reasons for Exercise**	**M**	**SD**	**M**	**SD**	**M**	**SD**	**M**	**SD**	**M**	**SD**	**F**	**eta**^**2**^
Mood enhancement	4.1	1.3	4.6	1.0	4.5	1.1	4.4	1.2	3.9	1.3	14.07***	.046 ^a,b,g,i,j^
Solitude	3.1	1.5	3.6	1.3	3.4	1.4	3.2	1.4	2.9	1.4	7.97***	.027 ^a,g,i^
Social	2.5	1.2	3.2	1.3	3.0	1.2	2.7	1.1	2.3	1.1	15.71***	.051 ^a,b,f,g,i^
Fitness	4.2	1.0	4.6	.80	4.6	.93	4.5	.86	4.0	1.0	16.59***	.054 ^a,b,c,g,i,j^
Preventative health	4.7	.96	4.7	1.1	4.8	1.0	4.6	.98	4.5	1.1	2.42**	.008
Weight management	3.9	1.3	3.8	1.2	3.8	1.2	3.8	1.3	3.8	1.4	.38	.001
Appearance	3.7	1.3	4.0	1.1	3.9	1.3	4.0	1.3	3.7	1.3	1.68	.006
Health concerns	3.3	1.3	3.1	1.3	3.1	1.3	3.3	1.3	3.0	1.3	1.74	.006
Competition	1.8	1.1	2.4	1.3	2.3	1.3	2.1	1.1	1.7	1.1	13.13***	.043 ^a,b,g,i^
**Motivation**	**M**	**SD**	**M**	**SD**	**M**	**SD**	**M**	**SD**	**M**	**SD**	**F**	**eta**^**2**^
External	1.6	.75	1.4	.57	1.6	.86	1.5	.72	1.6	.77	2.83**	.010 ^a,g^
Introjected	2.8	1.3	3.2	1.1	3.1	1.2	2.9	1.2	2.9	1.1	3.63***	.012 ^a^
Identified	3.8	.87	4.4	.58	4.3	.77	4.0	.81	3.7	.92	26.54***	.084 ^a,b,f,g,h,i,j^
Integrated	3.2	1.1	4.1	.97	3.8	1.1	3.4	1.1	2.9	1.2	32.37***	.100 ^a,b,f,g,h,i,j^
Intrinsic	3.4	1.1	4.0	.93	3.8	1.0	3.7	1.0	3.1	1.2	23.53***	.075 ^a,b,d,g,i,j^
Relative Autonomy	14.3	6.0	18.7	4.5	17.4	6.1	16.1	5.7	12.9	6.2	33.40***	.103 ^a,b,c,d,f,g,i,j^
**Physical Activity**	**M**	**SD**	**M**	**SD**	**M**	**SD**	**M**	**SD**	**M**	**SD**	**F**	**eta**^**2**^
Vigorous	86.6	86.1	441.1	131.3	249.1	158.0	172.2	153.2	90.5	96.2	321.16***	.525 ^a,b,c,e,f,g,h,i^
Moderate	95.1	85.1	156.1	109.2	390.1	121.8	180.7	149.1	94.3	104.3	216.12***	.426 ^a,b,c,e,g,h,i,j^
Walking	155.6	127.4	169.1	153.7	293.2	178.2	842.3	197.7	173.7	156.0	502.24***	.633 ^b,c,e,f,h,i, j^
Sitting	187.7	88.4	290.3	114.3	180.7	96.3	259.0	125.1	481.3	85.8	529.39***	.645 ^a,c,d,e,g,h,i^

Significant differences between profile groups are denoted by a = 1 vs. 2, b = 1 vs. 3, c = 1 vs. 4, d = 1 vs. 5, e = 2 vs. 3, f = 2 vs. 4; g = 2 vs. 5, h = 3 vs. 4, i = 3 vs. 5, j = 4 vs. 5. **p < 0.01. ***p < 0.001

**Fig. 1:**
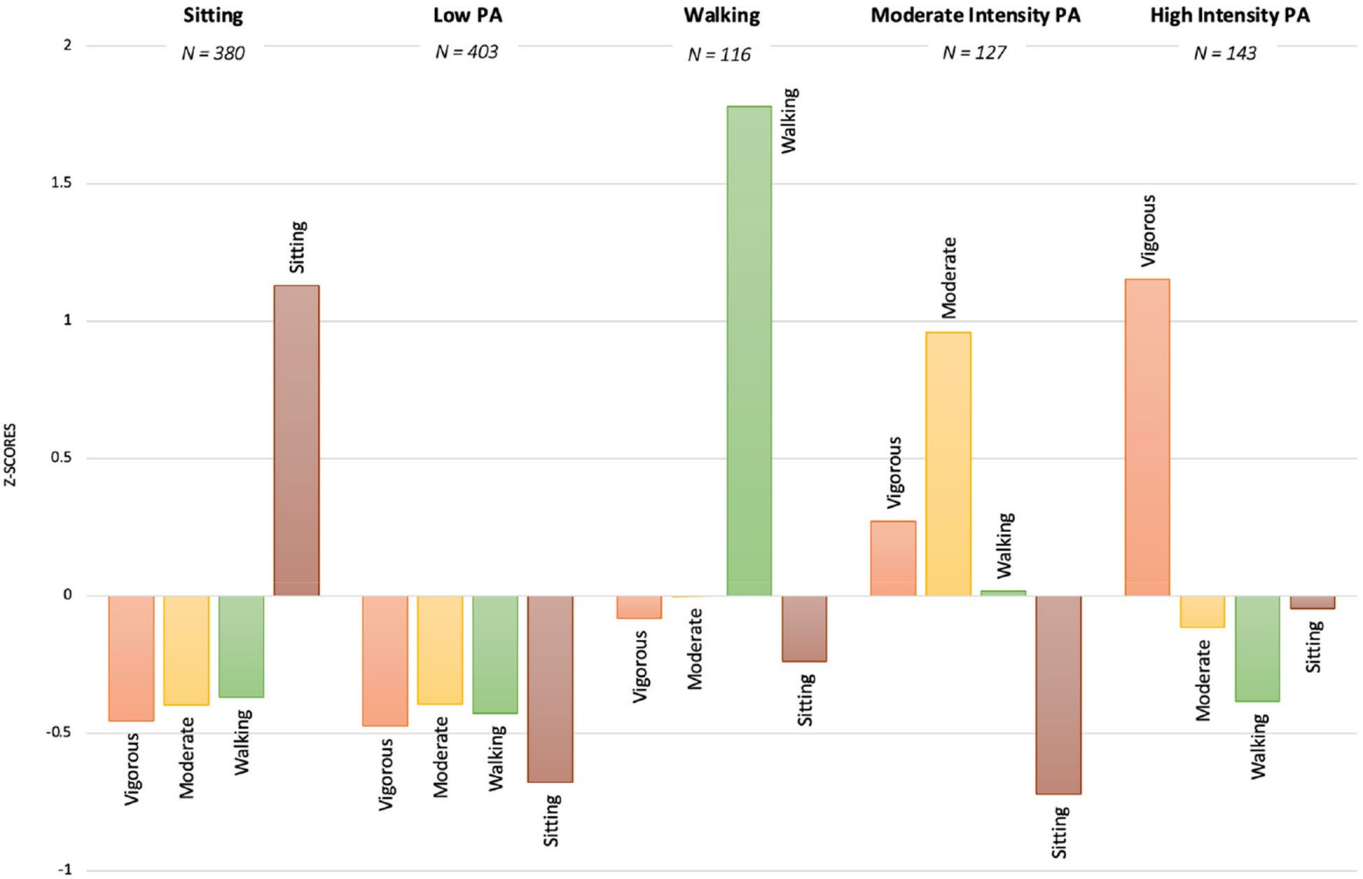
Five Profile solution Z-scores for the Physical Activity Clusters

The clusters were named based on the respondents’ participation in various PA intensity levels (see Fig. 1).

The total PA sum in minutes/week represented the combined total minutes spent in vigorous, moderate, and walking PA for the week reported for each cluster found in Table 3. Please refer to Table 3 for the total scores reported below for each cluster.

- *Cluster 1*. A “Sitting” profile (*n* = 380) included 32.5% of total participants, which represented the highest scores above the mean in time spent sitting compared to high and moderate intensity PA. The *Sitting PA* cluster engaged a combined total minutes spent in vigorous, moderate, and walking, representing an overall mean sum of 358.7 min/week (*SD* = 238.9).
- *Cluster 2.* A “Low” PA profile (*n* = 403) represented 34.5%, which was characterized by low scores below the mean in all PA intensity categories and engaged in a lower number of total PA minutes/week (i.e., total activity minutes in vigorous, moderate, and walking) for the week (M = 333.7.4; SD = 183.3; See Table 3). Compared to the sitting cluster, the low PA cluster reported a total mean of 187.7 min/week spent sitting whereas the sitting cluster had a mean of 481.3 min/week.
- *Cluster 3*. A “Walking” PA profile (*n* = 116) represented by 9.9% of the participants, which was characterized by high scores above the mean in walking compared to high and moderate intensity PA and sitting scores that were at or below the mean. The *Walking* PA cluster engaged in a combined total of 1195.3 PA minutes/week (*SD* = 344.3; see Table 3), which was signified to be the highest amount of total PA engagement compared to the other clusters.
- *Cluster 4.* A “Moderate Intensity” PA profile (*n* = 127) represented 10.9% of the participants characterized with high scores above the mean in moderate intensity PA compared to vigorous intensity PA scores slightly above the mean. The *Moderate Intensity* PA cluster engaged in a combined total of 932.5 min/week (*SD* = 297.9; see Table 3) of PA.
- *Cluster 5.* A “High Intensity” PA profile (*n* = 143) represented 12.2% of the participants due to high scores above the mean in vigorous intensity PA with scores below the mean in moderate intensity PA, walking, and sitting scores. The *High Intensity* PA cluster engaged in a combined total of 766.4 PA minutes/week (*SD* = 258.3; see Table 3).

### Differences between 5 PA profiles for reasons for exercise

**Fig. 2:**
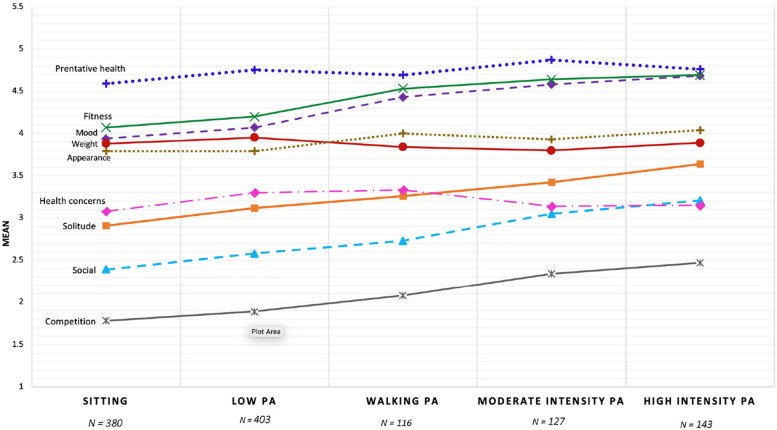
Mean scores of Reasons to Exercise subscales-based Z-scores for the 5 Physical

Profile differences were examined for reasons to exercise using MANOVA with univariate follow-up (see Table 3). Results showed a significant main effect, Wilks’ λ = 0.889; *F*(36, 4334) = 3.84, *p* < 0.001; partial *η*^2^ = 0.029. Across the 5-PA cluster profiles, five reasons showed significant differences, including: (a) mood enhancement, (b) solitude, (c) social, (d) fitness, and (e) competition (see Table 3 for detailed results and Fig. 2 for visual representation).

Bonferroni post-hoc tests suggested *High Intensity* PA cluster participants reported significantly higher scores for mood enhancement, solitude, social, fitness, and competition reasons for exercising compared to the *Low* PA and *Sitting* members. In all five of the PA profiles, there were no significant differences found for preventative health, weight management, appearance, or health concern reasons on any of the 5-PA profiles (see Table 3).

### Differences between five PA profiles for behavioral regulation

**Fig. 3:**
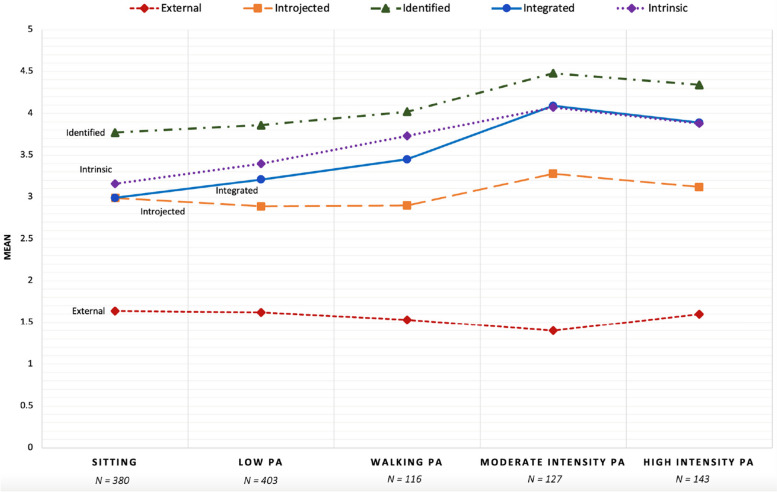
Mean scores of Motivation Regulation subscales based on the 5-solution for the Physical Activity Clusters

MANOVA results compared behavioral regulation subscales across the 5-profile PA solution and revealed significant multivariate associations (see Table 3). Results showed a significant main association, Wilks’ λ = 0.871; *F*(20, 3848) = 8.19, *p* < 0.001, partial *η*^2^ = 0.034. Specifically, follow-up ANOVA results indicated that all five of the behavioral regulation subscales and the RAI score revealed significant differences across the 5-cluster profile. According to Bonferroni post-hoc tests the *High Intensity* PA cluster reported significantly lower external regulation scores than all other clusters, with the *Sitting* cluster having the highest overall external regulation score. Additionally, compared to all other clusters, the *Sitting* profile demonstrated significantly lower scores on identified, integrated, intrinsic, and RAI scores (see Fig. 3).

Similarly, the *High Intensity* and *Moderate Intensity* PA clusters demonstrated significantly higher scores in identified, integrated, intrinsic, and RAI scores compared to the *Low* PA members. The *High Intensity* PA profile displayed significantly lower scores on external regulation compared to the *Sitting* and *Low* PA profiles. Results revealed a significant difference between the *Sitting* and *Low* PA profiles with *Low* PA demonstrating higher scores on intrinsic regulation.

Utilizing the RAI score as representative of motivational quality, the results revealed the unique magnitudes across the 5-PA clusters and represented higher relative autonomy from highest to lowest scores: *High Intensity*, *Moderate Intensity*, *Walking*, *Low*, and *Sitting* cluster (see Table 3). These results suggest that lower engagement in PA and sedentary behavior relate to motivation quality, and the higher the overall motivation quality the higher engagement in more high intensity physical activities.

## Discussion

The findings from this expand on previous research examining physical activity (PA) intensity profiles by creating and analyzing five unique PA engagement patterns (clusters) based on intensity levels. These clusters allow for a deeper understanding of how different intensities of PA relate to adult motivation and exercise goals (e.g., reasons for exercise).

### Physical activity cluster comparisons

The results comparing the PA profiles across adult’s exercise *goals*, which we refer to interchangeably as the reasons people have for exercising, supported significant and unique patterns. As hypothesized, the cluster analysis on PA categories was successful in creating unique profiles, with clusters differing on goals and behavior regulation subscales. Five unique PA clusters included the following: (1) a *Sitting* Profile – individuals in this cluster exhibited the highest levels of sedentary behavior; (2) a *Low* PA Profile – individuals in this cluster engaged in lower amounts of PA; (3) a *Walking* PA Profile – individuals in this cluster participated most in walking; (4) a *Moderate Intensity* PA Profile – individuals in this cluster engaged most in moderate PA; and (5) a *High Intensity PA* Profile – individuals in this cluster engaged most in vigorous PA It should be noted that the *Low* PA Profile does not indicate low levels of physical activity related to the U.S. Physical Activity Guidelines, rather the lower activity minutes with the exception of the sitting group, who also engaged in lower PA but was significantly more impacted with the highest reported levels of sitting behavior compared to the other profiles for the population included for this study.

### PA profile differences

Our analysis revealed that individuals in the *High Intensity* and *Moderate Intensity* PA clusters reported greater endorsement (i.e., highest scores) of exercise reasons related to mood enhancement, solitude, social, fitness and competition compared to those in more sedentary clusters, such as the *Sitting* and *Low* PA profiles. These results highlight the potential motivaitonal differences driving engagement across PA intensity levels. As expected, indivdiuals engaging in more vigorous PA tended to have higher intrinsic motivaiton as shown in their higher Relative Autonomy Index (RAI) scors. These findings support theories like the Dual-Mode Theory, suggesting that intrinsic motivations (e.g., enjoyment, mastery) become more prominent at higher intensities. In contrast, less physically active individuals (*Low* PA, *Sitting* profiles) scored higher on controlling forms of motivation (e.g., weight management, appearance), which have been associated with less autonomy and potentially less sustained PA engagement.

More specifically, our findings highlight that exercisers that engage in higher-intensity or moderate-intensity type of PA also appear to value solitude, social, fitness, and competition as important reasons for exercising more than those who exhibit more time spent engaging in higher sedentary behaviors. The *Low* PA cluster had lower scores on solitude, social, fitness and competition than did the *High Intensity* PA cluster, suggesting exercisers that participate in more intense levels of PA value these reasons. Compared to the *Walking* PA cluster, the *High Intensity* PA cluster reported significantly higher scores in social reasons for exercising (*M* = 2.3 vs. 3.2) highlighting that relatedness may play a positive and supportive role in promoting engagement in PA at higher intensities than lower intensity form of PA, such as walking. High intensity functional training, as a group exercise modality, and similar to other sports and group exercise forms, has been shown to elicit intrinsic motivation (i.e., enjoyment, challenge), competition, and relatedness factors [7, 35, 36]. The notion that participants engaging in higher intensity forms of PA also exhibited higher scores in solitude compared to the low PA group may shed light to a less understood area of research where both solitude and group-fitness may co-exist in strength-training focused programs such as in high intensity functional training (e.g., barbell and strength training) [7, 35]. While these programs may be group-focused, it also tends to be autonomous forms of exercise where the participant is responsible for lifting their own/individual weight (i.e., independence) despite being in a social, group-focused program. Further research in this area would be beneficial and help understand how to encourage participation in higher intensity PA, in which strong supports highlights the cardiometabolic benefits from such engagement [12, 37, 38].

Interestingly, no significant differences were found across the five PA clusters for reasons such as weight management, appearance, or preventative health. These more extrinsic goals may represent societal pressures that are broadly internalized across different activity levels but may not necessarily translate into consistent or vigorous PA engagement, aligning with past findings that these goals can be controlling forms of motivation.

For instance, these findings are aligned with previous work highlighting that for participants who frequently reported weight loss and health benefit focused goals as most important also reported the least amount of PA engagement overtime compared to other goals because, of the reasons why people adopt these particular types of goals [8, 9, 18, 21, 39]. These goals are suggested to be a controlling form of motivation [5, 22, 24, 40], associated with lower perceived autonomy due to the pressure to conform to societal/social pressures. Weight loss goals, particularly for aesthetic purposes, are often considered a controlling form of motivation. In contrast, health benefit goals are generally associated with identified regulation, reflecting an internalized motivation unless driven by external pressures such as recommendations from family or healthcare providers. As a result, pressures associated with health, do not to promote self-worth, have high psychological costs, and result in decreased autonomous states, and thus seen to be as relatively weak goals that are not essential, and therefore identifying patterns in PA is difficult [5, 18, 27, 40]. Another observation based on the study finding that individuals who considered the goals ‘weight loss’ and ‘heath benefits’ most important also reported the least amount of PA engagement over time may suggest that distal outcomes like weight management and preventative health, although important, may not provide immediate satisfaction that motivates sustained vigorous PA. In contrast, with the exception of 'fitness,' many of the reasons to exercise that had the strongest correlations with vigorous PA were those where the satisfaction of the outcome was immediate to the activity. These include goals such as mood enhancement, solitude, social interaction, and competition (see Table 1). This pattern is consistent with elements of Hedonic Theory, which posits that behaviors providing immediate pleasure and satisfaction are more likely to be sustained [41]. Therefore, the immediate benefits experienced during PA, rather than long-term health outcomes, may play a more critical role in maintaining high levels of engagement.

Consistent in the PA profile findings [7, 10, 36], it was evident that motivation may be critical to PA engagement, particularly in adults engaging in higher intensity PA. For example, compared to all the other clusters, the *High Intensity* PA profile contained higher scores on autonomous forms of motivational regulation subscales (i.e., identified, integrated, and intrinsic regulation) for exercising and lower scores on controlling forms of behavior regulation subscales (i.e., external and introjected regulation). Autonomy is instrumental in regard to adults engaging in higher intensity exercise [7, 36]. Additionally, the findings from this study highlight the value and importance in assessing the quality of motivation (i.e., RAI score). More specifically, the *High Intensity* cluster had the highest RAI score followed by the *Moderate Intensity* and *Walking* PA clusters, confirming our hypotheses that not only is motivation important in more intense forms of PA, but the motivation quality may also be critical. Findings could be explained by the Dual-Mode Theory described by Ekkekakis [41] which postulates cognitive variables such perceived efficacy, goals, and expectations are more important as high intensity exercise levels are approached [42]. As perceived efficacy and reasons to exercise are heightened during high intensity activity, then autonomous motivation for exercise could increase, impacting the RAI score [9]. Enjoyment, mastery, and self-induced challenges were predicted more by intrinsic motivation in moderate and vigourous intensity exercisers, suggesting a higher RAI score in higher intensity exercisers. Further confirmations of these findings are best highlighted in Box, Feito, Brown, Heinrich, and Petruzzello in [7] suggesting that high intensity PA, similar to group exercise and sports, elicits intrinsic motivaiton (i.e., enjoyment, challenge), compeition, and relatedness factors.

Furthermore, the relatively low percentage of individuals falling into the *High Intensity* PA profile (~ 12% of the sample) reinforces the challenge of fostering vigorous PA engagement in the general population. Similarly, the *Moderate Intensity* PA profile represented ~ 11%, and the *Walking* profile included 10% of the population, whereas ~ 68% of the participants represented either the *Low* PA or *Sitting* profile. The *Low PA profile* had the lowest score in PA intensity categories and the lowest number of total PA per week. The *Sitting* profile represented the highest scores above the mean in time sitting across the groups. It is paradoxical that despite the inclusion criteria of this study being that participants must be engaging in PA or planning to start/restart PA, the *sitting* profile was the most prominent among an active population. This indicates that among a highly active group, individuals might not be engaging in as much activity as they might perceive. These findings also align with the prevalence of high sitting time and physical inactivity among U.S. adults. About 1 in 4 adults sit for more than 8 h a day, 3 in 4 are physically inactive, and 1 in 10 report both [43]. This higher level of low activity and sitting is even seen in a more highly active group. Evidence-based strategies to reduce sitting time, increase physical activity, or both would potentially benefit most sedentary U.S. adults.

Overall, our findings suggest a complex, bidirectional relationship between goals, motivation, and PA behavior. While initial goals may influence PA engagement, the intensity and type of PA can also shape and refine an individual's goals and motivations over time. This highlights the importance of creating environments that not only encourage goal setting but also provide positive, intrinsically motivating PA experiences to sustain long-term engagement.

### Summary

The study revealed distinct differences across physical activity (PA) profiles, with the High Intensity and Moderate Intensity PA clusters scoring the highest on mood enhancement, solitude, social, fitness, and competition reasons for exercise, significantly higher than sedentary participants. Interestingly, high-intensity exercisers valued both solitude and social connection, particularly in strength-training programs that emphasize autonomy within group settings. No differences were found across PA clusters for goals related to preventative health, weight management, appearance, or health concerns, suggesting these may be culturally shared forms of motivation. The findings also indicated that immediate satisfaction from PA-related outcomes, such as mood enhancement and social interaction, played a more critical role in maintaining high levels of PA compared to distal health goals. Autonomous motivation was highest in the High Intensity PA cluster, as evidenced by the highest Relative Autonomy Index (RAI) scores, underscoring the role of autonomy in sustaining high-intensity exercise. Finally, the majority of participants (68%) fell into the Sitting or Low PA profiles, highlighting the need for strategies to increase PA engagement and reduce sedentary behavior, even among those who identify as physically active.

#### Strengths, limitations, and future directions

The present study has important strengths, limitations, and implications to highlight. One primary strength was the large research sample studied, consisting of adults (e.g., motivated respondents) varying in age levels from 18 to 87 years of age. A future research direction would be to explore the mediating or moderating factors that influence the motivation around reasons for exercise. It may also be of interest to explore demographic differences within the clusters and how social factors might drive reasons for activity. The impact of behavior-change interventions encouraging exploration of values through practices like health coaching and peer mentorship might also be a promising research direction building on supportive work from researcher findings [44, 45].

As with any study, there were limitations in this research. First, the design of the study is cross-sectional and utilizes a convenience sample. However, the sample size and the participants recruited highlight that even exercisers perceived to be highly physically active may not be as active as health professionals believe them to be. Therefore, it is not possible to infer causal relationships from the results. Second, PA was assessed using a self-report measure. The limitations of this study include possible bias inherent in self-reported data and that physical activity episodes shorter than 10 min may not have been captured. Lastly, it must be emphasized that the subscales utilized in the Reasons to Exercise Scale (RE_X_) are most likely not the only reasons adults have for exercising and/or being physically active. It should be noted that these motivation factors cannot be generalizable to those who are not physically active.

##### Practical implicaitons

The results of this study have several practical implications for public health interventions aimed at increasing PA engagement across populations. First, interventions that promote high-intensity PA should focus on intrinsic motivation by creating environments that foster enjoyment, social interaction, and a sense of personal accomplishment. For example, group-based high intensity training programs that balance social interaction with autonomy (e.g., individualized challenges within a group setting) may be particularly effective in promoting sustained engagement.

In addition, given that many participants across clusters still reported extrinsic goals such as weight management and health benefits, interventions should aim to reframe these goals in a more autonomous manner. For instance, encouraging participants to connect these types of external goals to personal values (e.g., improving health for long term wellbeing rather than appearance) may enhance their sense of ownership and autonomy, potentially leading to more sustained PA participation.

Lastly and most importantly, an essential aspect of interpreting these findings is that future research considers how motivation constructs can be adopted and integrated into interventions.

The Capability, Opportunity, and Motivation Behavior (COM-B) model offers a useful framework for understanding behavior change, positing that behavior results from the interaction of capability (physical and psychological), opportunity (physical and social), and motivation (reflective and automatic) [46, 47]. Applying the COM-B model in future research could clarify how motivations influence PA behavior and guide the design of interventions to boost engagement [48]. For instance, improving physical capability through strength training, enhancing psychological capability via self-efficacy, and fostering supportive social environments and intrinsic motivation could help sustain PA over time.

###### Future research directions

Future research should continue to investigate the interplay between PA intensity, motivation quality, and goals to better understand how these factors influence long-term PA engagement. Longitudinal studies that track changes in motivation and goal orientations over time as individuals progress through different stages of PA engagement would provide valuable insights into how PA can be sustained. Additionally, interventions that explicitly target the enhancement of intrinsic motivation (e.g., through autonomy-supportive environments) should be explored to determine their effectiveness in promoting higher intensity PA, especially among sedentary or minimally active populations.

Lastly, further exploration of the potential paradox between the sedentary behaviors observed in this study and participants' reported intentions to engage in PA could shed light on barriers to PA engagement and inform strategies for reducing sitting time in both recreational and everyday contexts.

## Supplementary Information

Supplementary Material 1.

## Data Availability

Data is available upon request from corresponding author.

## Abbreviations

PA: Physical Activity
IPAQ: The International PA Questionnaire
RE_X_-2: Reasons to Exercise Scale 2
BREQ-3: Behavioral Regulation in Exercise Questionnaire-3
MANOVA: Multivariate analysis of variance
SDT: Self-Determination Theory
MPAM: Motives for Physical Activity Measure
MPAM-R: Motives for Physical Activity Measure-Revised
PALMS: Physical Activity Leisure Motivation Scale
SPSS: The Statistical Package for the Social Science
RAI: Relative Autonomy Index
SD: Standard Deviation
COM-B: The Capability, Opportunity, and Motivation Behavior
