# Supplementary material for: A profile approach to physical activity levels: what’s intensity got to do with reasons and motives for exercise?
Source: BMC Public Health. 2024 Oct 29;24:2990. doi: 10.1186/s12889-024-20449-1 (PMC11520888; doi:10.1186/s12889-024-20449-1)
Supplement: Supplementary file 1 — Supplementary Material 1. [file 12889_2024_20449_MOESM1_ESM.docx]

**Supplemental Table 1.** Descriptives and Correlations for Physical Activity Intensity Levels, Motivation Regulation, and RAI Scores.

|  | **Variable** | **1** | **2** | **3** | **4** | **5** | **6** | **7** | **8** | **9** | **10** | **11** | **12** | **13** | **14** | **15** | **16** | **17** | **18** | **19** |
| --- | --- | --- | --- | --- | --- | --- | --- | --- | --- | --- | --- | --- | --- | --- | --- | --- | --- | --- | --- | --- |
| **PA Levels** | 1. Vigorous PA | -- |  |  |  |  |  |  |  |  |  |  |  |  |  |  |  |  |  |  |
|  | 2. Moderate PA | .30 | -- |  |  |  |  |  |  |  |  |  |  |  |  |  |  |  |  |  |
|  | 3. Walk PA | .11 | .22 | -- |  |  |  |  |  |  |  |  |  |  |  |  |  |  |  |  |
|  | 4. Sitting | -.15 | -.18 | -.09 | -- |  |  |  |  |  |  |  |  |  |  |  |  |  |  |  |
| **Reasons for Exercise** | 5. Mood enhancement | .31 | .19 | .09 | -.12 | (.91) |  |  |  |  |  |  |  |  |  |  |  |  |  |  |
|  | 6. Solitude | .27 | .14 | .09 | -.13 | .62 | (.93) |  |  |  |  |  |  |  |  |  |  |  |  |  |
|  | 7. Social | .29 | .15 | .07 | -.13 | .48 | .39 | (.90) |  |  |  |  |  |  |  |  |  |  |  |  |
|  | 8. Fitness | .32 | .13 | .11 | -.13 | .56 | .36 | .42 | (.85) |  |  |  |  |  |  |  |  |  |  |  |
|  | 9. Weight management | **.01^#^** | **-.05^#^** | **-.01^#^** | **-.01^#^** | .19 | .15 | .15 | .26 | (.90) |  |  |  |  |  |  |  |  |  |  |
|  | 10. Preventative health | .11 | **.06^#^** | **.04^#^** | -.10 | .39 | .26 | .23 | .57 | .30 | (.86) |  |  |  |  |  |  |  |  |  |
|  | 11. Appearance | .12 | **.04^#^** | **.03^#^** | **-.02^#^** | .40 | .24 | .23 | .42 | .59 | .30 | (.94) |  |  |  |  |  |  |  |  |
|  | 12. Health concerns | **-.04^#^** | **-.01^#^** | **.04^#^** | -.06 | .06 | .11 | .10 | .17 | .30 | .24 | **.05^#^** | (.84) |  |  |  |  |  |  |  |
|  | 13. Competition | .30 | .17 | .08 | -.11 | .33 | .33 | .51 | .38 | **.04^#^** | .10 | .27 | -.06 | (.93) |  |  |  |  |  |  |
| **Motivation** | 14. External | .09 | **.02^#^** | **-.01^#^** | **-.02^#^** | **.02^#^** | **-.01^#^** | -.12 | **-.04^#^** | -.23 | -.08 | -.17 | -.20 | -.12 | (.83) |  |  |  |  |  |
|  | 15. Introjected | -.17 | **-.04^#^** | **.01^#^** | **-.01^#^** | -.37 | -.25 | -.20 | -.35 | -.30 | -.22 | -.42 | **-.01^#^** | -.24 | .25 | (.89) |  |  |  |  |
|  | 16. Identified | .40 | .22 | .07 | -.13 | .63 | .42 | .36 | .57 | .08 | .43 | .28 | **-.02^#^** | .28 | **.06^#^** | -.50 | (.81) |  |  |  |
|  | 17. Integrated | .43 | .22 | .09 | -.16 | .59 | .40 | .45 | .57 | **.04^#^** | .40 | .28 | **-.05^#^** | .39 | **.03^#^** | -.45 | .80 | (.90) |  |  |
|  | 18. Intrinsic | .38 | .22 | .10 | -.17 | .69 | .48 | .46 | .47 | **.01^#^** | .27 | .21 | -.08 | .35 | .12 | -.28 | .71 | .71 | (.94) |  |
|  | 19. RAI Score | .42 | .24 | .10 | -.18 | .64 | .44 | .42 | .49 | -.08 | .30 | .15 | -.12 | .31 | .38 | -.16 | .77 | .82 | .91 | -- |
|  | *Mean* | 157.4 | 142.8 | 246.2 | 302.1 | 1.6 | 3.0 | 3.9 | 3.3 | 3.5 | 15.0 | 3.9 | 3.2 | 2.0 | 1.6 | 3.0 | 3.9 | 3.3 | 3.5 | 15.1 |
|  | *SD* | 163.2 | 140.1 | 253.8 | 161.2 | 1.3 | 1.4 | 1.2 | 1.0 | 1.3 | 1.0 | 1.3 | 1.4 | 1.2 | 1.3 | 1.4 | 1.2 | 1.0 | 1.3 | 6.2 |

Note. All correlations significant at p ≤ 0.05 unless otherwise bolded and ^#^ p > 0.05. SD = standard deviation; RAI = Relative Autonomy Index score (i.e., motivation quality); PA = Physical Activity. Values in parantheses represent the cronbach’s alpha score for the Reasons to Exercise Scale (RE_X_) and Motivation Regulation (BREQ-3) subscales.
